# Implantable porous gelatin microspheres sustained release of bFGF and improved its neuroprotective effect on rats after spinal cord injury

**DOI:** 10.1371/journal.pone.0173814

**Published:** 2017-03-14

**Authors:** Li Lan, Fu-Rong Tian, De-Li ZhuGe, Qi-Chuan ZhuGe, Bi-Xin Shen, Bing-Hui Jin, Jian-Ping Huang, Ming-Ze Wu, Lu-Xin Fan, Ying-Zheng Zhao, He-Lin Xu

**Affiliations:** 1 Zhejiang Provincial Key Laboratory of Aging and Neurological Disorder Research, First Affiliated Hospital of Wenzhou Medical University, Wenzhou, China; 2 School of Pharmaceutical Sciences, Wenzhou Medical University, Wenzhou City, Zhejiang Province, China; 3 WenZhou Chinese Medicine Hospital, WenZhou, Zhejiang Province, China; University of Toronto, CANADA

## Abstract

In this study, porous gelatin microspheres (GMSs) were constructed to improve the neuroprotective effect of basic fibroblast growth factor (bFGF) on spinal cord injury. GMSs were prepared by a W/O emulsion template, followed by cross-linking, washing and drying. The particle sizes and surface porosity of the blank GMSs were carefully characterized by scan electronic microscopy. The blank GMSs have a mean particle size of 35μm and theirs surface was coarse and porous. bFGF was easily encapsulated inside the bulk GMSs through diffusion along the porous channel. 200μg of bFGF was completely encapsulated in 100mg of GMSs. The bFGF-loaded GMSs displayed a continuous drug release pattern without an obvious burst release over two weeks in vitro. Moreover, the therapeutic effects of bFGF-loaded GMSs were also evaluated in spinal cord injury rat model. After implantation of bFGF-loaded GMSs, the recovery of the motor function of SCI rats were evaluated by behavioral score and foot print experiment. The motor function of SCI rats treated with bFGF-loaded GMSs was more obvious than that treated with free bFGF solution (P<0.05). At the 28^th^ days after treatment, rats were sacrificed and the injured spinal were removed for histopathological and apoptosis examination. Compared with treatment with free bFGF solution, treatment with bFGF-loaded GMSs resulted in a less necrosis, less infiltration of leukocytes, and a reduced the cavity ratio and less apoptotic cells in injured spinal(*P*<0.01), indicating its better therapeutic effect. Implantable porous GMSs may be a potential carrier to deliver bFGF for therapy of spinal cord injury.

## Introduction

Spinal cord injury (SCI), one of the most devastating traumas, leads to sudden loss of sensory, motor, and autonomic function distal to the level of injury [1]. The pathology of SCI is divided into two stages. The primary injury is the mechanical impact afflicted directly on the spine. The secondary injury is a complex cascade of molecular events including disturbances in ionic homeostasis, local edema, ischemia, focal hemorrhage, oxidative stress and inflammation, with apoptosis playing a vital role in the progressive degeneration of injured spinal cord. Basic fibroblast growth factor (bFGF) has been shown to inhibit apoptosis of nerve cells and exhibit neuroprotective effect [2]. bFGF has been used to treat stroke and SCI animals, showing a good therapeutic effect[3]. For example, the delivery of bFGF using an osmotic minipump in vivo has demonstrated improvement of functional recovery in SCI rat model [4, 5]. Inversely, systemic delivery is not suitable for b-FGF. First, bFGF is a biological macromolecular protein, if systemic administration is likely to be related to enzymes degradation, and not easy to pass through the blood spinal cord barrier. Besides, its mitogenic potential may lead to malignant tumors in healthy tissues. To overcome the shortcomings of protein-based therapeutics, an in situ drug delivery system is effective and safe strategy for local delivery of these therapeutic agents to the injured site. Gelatin is nontoxic, biodegradable, and can act as a protecting agent and depot preparations. For example, a recent study demonstrated that tracheal cartilage is regenerated by the slow release of bone morphogenetic protein or bFGF from gelatin sponge through local implantation [5, 6]. Gelatin microspheres are also widely accepted as drug carriers for local implantation to deliver various growth factors, such as bone morphogenetic protein 2, vascular endothelial growth factor and bFGF, stem cell and plasmids into tissues of the body to facilitate tissue regeneration and remodeling [7–10].

In this study, a porous gelatin microsphere (GMSs) was prepared by a template method to encapsulation of bFGF. The bFGF-loaded GMSs were implanted to the injured spinal zone for sustained release of bFGF. The neuroprotective effect of bFGF-loaded GMSs on SCI rats were evaluated by behavioral score and foot print experiment. Moreover, the therapeutic effect of bFGF-loaded GMSs was also evaluated by the histopathological and apoptosis examinations.

## Materials and methods

### Details about the care and use of animals

Animal studies were performed with the approval from the ethical committee of Wenzhou Medical University and experiments were performed according to the National Institutes of Health Guide for the Care and Use of Laboratory Animals, so animals received high level of humane care. A standard commercial rat chow and water were available ad libitum Animal Use Permit No.:SYXK(Zhe)2010-0150; Animal experimental training certificate:X1504077. Sprague-Dawley rats (250 ± 20g, female, Slac Laboratory Animal Corporation, Shanghai, China) were housed at 25 ±2°C and humidity of 50±10% controlled with a 12 h light/12 h dark cycle, and given free access to water and food. Two to three animals were housed per stainless steel cage on a 12h light/12h dark cycle in an air-conditioned room at 22°C, and check daily by the animal care staff. Before the experiment, the behavior of all rats was detected for adapting to the experiment detection. All the animals were anaesthetized by an intraperitoneal injection of pentobarbital sodium (60 mg/kg), and then placed on a constant temperature heating platform. Then surgical procedures were performed on deeply anesthetized animals. After the experiment, the animals were kept in a cage to avoid cross effects. After surgery, rats were returned and received manual bladder expression twice daily until bladder function was restored. Before the animal tissues were acquired, rats were euthanasia by an intraperitoneal injection with pentobarbital sodium. The dose of euthanasia is about 3 times as much as the amount of narcotics. Criteria for judging the death of animals is continuous no spontaneous breathing for 2-3min and no blink reflex. The university will make a unified approach to the management of experimental animals in accordance with the ethics committee.

### Reagents and antibody

All reagents used in this study were commercially available. Gelatin A with an isoelectric point of 5.0 was isolated from bovine bone collagen by an alkaline process (Sigma-Aldrich). Basic fibroblast growth factor (bFGF) with an isoelectric point of 9.6 was ordered from Gelusite Biology Technology Company, Zhejiang, China. Rabbit polyclonal anti-caspase-3, rabbit monoclonal anti-neuron, and rabbit polyclonal anti-GFAP, and chicken polyclonal anti-Neurofilament (NF200) were purchased from Abcam (Abcam, CB, UK). A donkey polyclonal anti-rabbit IgG-HRP (Abcam, CB, UK) was used as the secondary antibody. Fluorescein isothiocyanate (FITC) was ordered from Sigma (Sigma-Aldrich, St. Louis, MO, USA).

### Preparation of porous gelatin microspheres

Gelatin microspheres were prepared by w/o emulsion crosslinking reaction as previously described [11], but with some modification. Briefly, 15% (w/v) of gelatin solution was firstly prepared, and then 0.2g of NaCl was dissolved in 4ml of gelatin solution using as aqueous phase. Afterward, the aqueous solution was added dropwise into 40 ml of paraffin solution containing 1.5% spann-80 preheated to 50°C. The two phases were emulsified for 30min at 800rpm using paddle stirring. The w/o emulsion was then cooled in ice bath and stirring continued for 30min to allow the spontaneous gelation of gelatin aqueous solution. Followed by, 40 ml of paraformaldehyde/isopropanol (5:35, v/v) solution was added to the cooled o/w emulsion, adjusted pH of reaction mixture to 9.0 using NaOH solution and stirring continued for 3h to crosslink the microspheres. The microspheres were collected by centrifugation at rate of 3000rpm, washed several times with isopropanol to remove residual oil from their surface and dried for one night in a vacuum. Finally, the microspheres were washed with distilled water for thrice times to remove the porous-forming agent, NaCl, and dried again in a vacuum. As a control, normal microspheres were prepared by the similar procedure above just without addition of porous-forming agent, NaCl.

### Preparation of bFGF-loaded gelatin microspheres

bFGF-loaded gelatin microspheres were prepared according to the method described in literature[10], but with some modification. Briefly, bFGF was encapsulated into gelatin microsphere by adding an aqueous solution of bFGF (0.3mg/mL) to 100mg of dry gelatin microspheres, and then allowing the suspension to stand for 4 h at room temperature. Subsequently, the bFGF/gelatin microspheres mixture was washed with 300μL of distilled water two times to remove non-encapsulated bFGF, and the bFGF-loaded gelatin microspheres were collected and lyophilized. The lyophilized bFGF-loaded microspheres were further annealed by incubation with 10% of human serum albumin at 37°C for 12h, and then collected and lyophilized. Just prior to injection, sterile phosphate buffer solution (PBS, pH 7.4) was added to dry bFGF-loaded microspheres, making a 50μL of microspheres suspension for each rat. As a control, unloaded dry microspheres were hydrated by adding sterile PBS.

### Encapsulation efficiency

Encapsulation efficiency was determined by using an ELISA kit (Sigma-Aldrich). In brief, an aqueous solution of bFGF (0.3mg/mL) was added to dry gelatin microspheres at various ratios (50, 100, 200, 300, 500μg of bFGF to l00mg of dry gelatin microspheres and then allowing the suspension to stand for 4 h at room temperature. Subsequently, bFGF/gelatin microspheres mixture was washed with 300μL of distilled water two times to remove non-encapsulated bFGF and supernatants were collected after each washing. After centrifuging at 2000 rpm for 2 min, the supernatant was withdrawn and amounts of bFGF in the total collected supernatant were measured using the ELISA kit. The loading efficiency of bFGF-loaded gelatin microspheres was calculated using the following formula: Loading efficiency (%) = (total bFGF-bFGF in supernatant)/total bFGF×100. Three replicates of each sample were analyzed.

### Characterization of microspheres

#### Particle size and morphology of gelatin microspheres

Sphericity and particle size were determined by measuring the diameter of individual particles using an optical microscope with micrometer (2XC, Shanghai, China). The diameters of 500 particles were measured and the mean particle size determined. The size distribution was calculated by SPSS13.0 statics software.

The size distribution and surface morphology of microspheres were further examined on a scanning electronic microscope (Hitachi, Japan). The drug-loaded microspheres were positioned on a mental stud, which was coated with an adhesive label. The different magnification images were captured by adjusting the voltage.

#### In vitro release from bFGF-loaded microspheres

FITC-labeled bFGF (FITC-bFGF) replaced bFGF to prepare the FITC-bFGF-loaded gelatin microspheres for the in vitro release study. The method for the in vitro release of FITC-bFGF was referred to the previous reports in the literature [12] with some modifications. Briefly, 50mg of FITC-bFGF-loaded gelatin microspheres sample was dispersed in 1.5 mL of PBS and placed in thermostatic oscillator. At established time intervals, the microspheres sample was centrifuged at a speed of 3000 rpm for 5 min, and 150μL of the supernatant was collected and replaced with equal volume of fresh PBS to maintain a constant volume. The fluorescence intensity of samples at different time points (I_t_) was quantified using a Thermo Scientific Microplate Reader at λ_ex_ = 495 nm and λ_em_ = 525 nm. The total fluorescence intensity (I_total_) of the encapsulated FITC-bFGF was referred as the fluorescence intensity value of FITC-bFGF solution, prepared by dissolving equivalent amount of FITC-bFGF in the same volume of released medium. The in vitro cumulative release rate of FITC-bFGF from the FITC-bFGF-microspheres was calculated according to the following formula.

$$\text{Cumulative release rate }(\%)=\frac{\sum I_{t}}{I_{total}}\times 100\%$$

### Animal model of SCI and group of drug administration

Healthy female adult Sprague-Dawley (SD) rats with a body weight of 240–260 g were used for surgical procedures. All the animals were anaesthetized by an intraperitoneal injection of pentobarbital sodium (50 mg/kg). After that, rats were placed on a constant temperature heating platform. The rats were placed in prone position, and fixed on the ridge fixation (attached to LISA instrument for spinal cord injury model, Louisville Impactor System Apparatus, Inc,Louisville,KY) [13,14].The backs of rats were shaved and muscles above the midline were softly removed at thoracic level. A laminectomy was performed on the thoracic vertebras 9–10 (T9–T10). The rat T9 dorsal midline was used as a reference. An incision was made with vibraknife at LISA instrument. Then a hemisection was introduced at the right side of the spinal cord, sparing the left section only [15, 16]. The spinal cord volume of the defect is 5mm×1.5mm×2mm. Sham group animals received the same surgical procedures without the resected injury of spinal. All animals were divided into different groups randomly. bFGF solution/ bFGF-loaded microspheres suspension were injected into the lesion at a dose of 20 μl (60μg bFGF/each rat) through a 16-gauge needle. The rats of sham group and SCI group were administered with same dose saline. After surgery, rats were returned and received manual bladder expression twice daily until they restore bladder function.

### Basso, Beattie, and Bresnahan (BBB) scoring and footprint analysis

Basso, Beattie, and Bresnahan (BBB) scoring was a common method to assess the locomotor function of SCI rats after different treatment. Two trained investigators who were blind to the experimental conditions scored the locomotion recovery in an open field according to BBB scale. BBB score ranged from zero point, indicating no observed hind limb movements, to 21 points, representing a normal ambulating rodent. Rats were placed individually on open fields and allowed to move freely for 5 min. The BBB score was evaluated at 1d, 3d, 7d, 14d and 28d for each group after different treatment.

Footprint analysis was performed through dipping the animal’s hindpaws in dye [17]. All rats were allowed to walk across a narrow box (1 m long and 7 cm wide). The footprints were scanned and digitized images were analyzed.

### HE staining of spinal cord

At 28 days after treatment, the rats were anesthetized by intraperitoneal injection with 1% of pentobarbital sodium solution (50 mg/kg) and perfused with 0.9% NaCl. The spinal cords from the T8–T10 level were excised and stored in cold 4% paraformaldehyde overnight, then embedded in paraffin. Longitudinal sections (5μm thickness) of the embedded spinal cord were mounted on poly-L-lysine-coated slides and stained with haematoxylin and eosin (HE) before observation under the light microscope.

### Immunohistochemical staining

The level of protein expressed in each experimental group was detected by immunohistochemical staining. The paraffin sections (5μm thickness) of the spinal cords were incubated with the primary antibody (Primary antibody: polyclonal rabbit anti-rat caspase-3 antibody (1/100), monoclonal rabbit anti-rat neuron antibody (1/3000), and polyclonal rabbit anti-rat glial fibrillary acidic protein antibody (GFAP, 1/5000) and polyclonal chicken anti-Neurofilament heavy polypeptide antibody (NF200, 1/5000)) at 4°C overnight, then washed with PBS three times. The paraffin sections were incubated with horseradish peroxidase-conjugated secondary antibody for 2 h at 37°C (Second antibody: A donkey polyclonal anti-rabbit IgG-HRP (1/500) was used as the secondary antibody; A donkey polyclonal to chicken IgY-Alexa Fluor488 (1/100) was used as the secondary antibody). The reaction was stopped with 3, 3-diaminobenzidine (DAB). The results were imaged using an optical microscope (Nikon ECLIPSE Ti-S, Ruikezhongyi Company, Beijing, China). The total number of staining-positive cells in each representative mesencephalic section was counted for the striatum region by technicians who were blind to the treatment. The quantification of each density was performed using the Image-Pro Plus software.

### TUNEL apoptosis assay

DNA fragmentation in vivo was detected using a one-step TUNEL Apoptosis Assay KIT (Roche, Mannheim, Germany). The images were captured with a Nikon ECLIPSE Ti microscope (Nikon, Japan).

### Statistical analysis

All statistics were performed using one-way analysis of variance (ANOVA) test, followed by Dunnett’s post hoc test for more than two groups. Statistical significance was determined with a Student’s t test for two experimental groups Differences were accepted to be statistically significant at values of P<0.05. The data were expressed as the mean ±SEM.

## Results and discussions

### Morphology and particle size of porous gelatin microspheres

**Fig 1:**
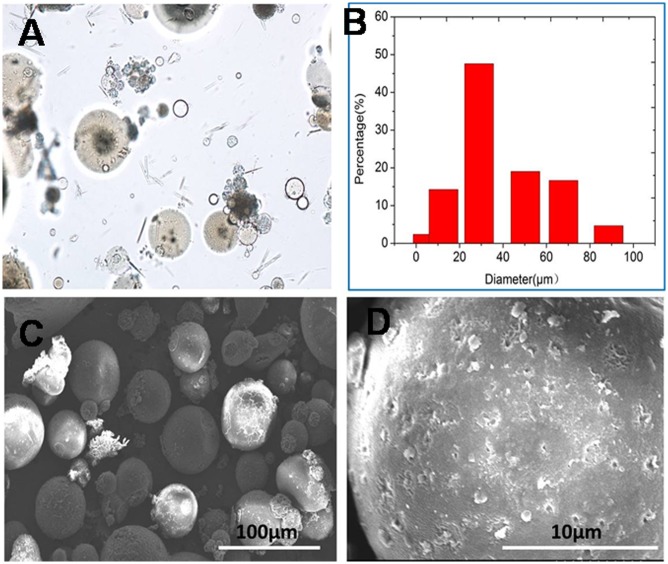
In vitro characteristics of porous gelatin microsphere. (A) spherical morphology of porous gelatin microsphere under optic microcopy, (B) particle size distribution of porous gelatin microspheres, (C) morphology of porous gelatin microsphere under scanning electronic microscopy and (D) the porous surface of porous gelatin microspheres.

The shape and particle size of a carrier for bioactive molecules are two important parameters to tightly control over the physicochemical properties of a drug delivery system such as its loading efficiency, and its ability of the sustained-release of its payload. In this study, a porous gelatin microsphere was prepared by s/o emulsion crosslinking method using NaCl as porous-forming agents. Morphology and Particles size of porous microspheres were shown in Fig 1. Although there existed some gelatin fragment in microspheres samples, most of microspheres were spherical shape and their particle sizes ranged from 5μm to 86μm, having a mean particle size of 35μm (Fig 1A, 1B and 1C). Before washing with water, the surface of microsphere was smooth and dense, without any porosity (S1 Fig). However, the surface of microspheres was coarse and porous, and the pore size was about 0.5μm (Fig 1D). Porosity of microsphere was caused by dissolving NaCl embed in microsphere during washing with water. The porous surface was highly valuable for drug loading in free organic solvent medium. As a control, the normal gelatin microsphere was also prepared without addition of NaCl. Whatever washing with water or not, the surface of normal gelatin microspheres was of little porosity (S2 Fig).

### Encapsulation of bFGF in porous microspheres

**Fig 2:**
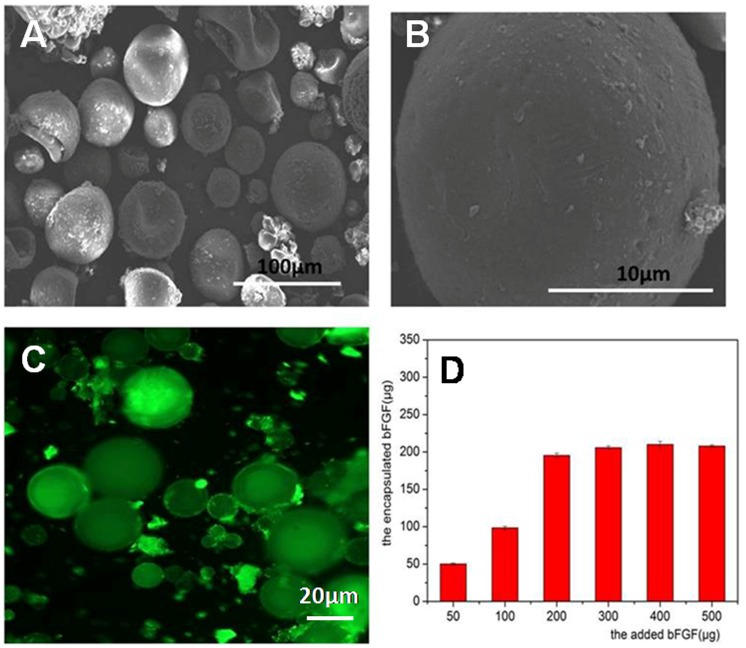
(A) SEM morphology of bFGF-loaded gelatin microsphere, (B) surface of bFGF-loaded gelatin microsphere observed by scanning electronic microscopy, (C) morphology of FITC-bFGF loaded microsphere under fluorescence microscopy and (D) the relationship between amount of bFGF encapsulated in porous gelatin microspheres and the added bFGF.

bFGF was susceptible to be denatured when exposing to organic solvents, high temperature and harsh pH. bFGF was loaded inside porous microsphere by dropping 1mg/mL of bFGF solution into freeze-dried gelatin microspheres, followed by annealing microsphere with human serum albumin. The morphology of bFGF-loaded microspheres was shown in Fig 2A and 2B. Bulk morphology of bFGF-loaded microspheres did not changed, but the porosity on microsphere surface disappeared after loading bFGF, indicating the efficient loading of bFGF. To ascertain the maximum loading amount of bFGF, various amount of bFGF were added to 100mg of freeze-dried microspheres for 12h of incubation. And then, the practical bFGF encapsulated in microspheres was determined and results were shown in Fig 2C. As the amount of the added bFGF increased, the encapsulated bFGF increased accordingly but a maximum amount, 200μg of bFGF loaded per 100mg of microspheres, was reached when 200μg of bFGF was added. In order to investigate the distribution of the encapsulated bFGF inside microspheres, FITC labeled bFGF (FITC-bFGF) replaced native bFGF and were encapsulated into porous gelatin microsphere by the same procedure. And the fluorescence image of FITC-bFGF-loaded microspheres was taken and result was shown in Fig 2D. The green fluorescence was uniformly distributed inside the bulk microspheres, indicating bFGF permeated the intact microsphere not just distributed on surface of microspheres. Two aspects of reasons may involve in the strong and uniform distribution of FITC-bFGF. Firstly, the porous network of porous GMSs may be an important factor, which resulted in the homogenous drug distribution in bulk microspheres, because normal microspheres without porous surface only exhibited a strong fluorescence on its surface after loading FITC-bFGF (S2 Fig). Second, the strong electrostatic interaction between acid gelatin and basic bFGF inside microspheres was also a dominate reason. When the porous GMSs was incubated with free FITC and then observed by fluorescent microscopy, the fluorescent of GMSs was very weak and only distributed on its surface (S3 Fig), indicating the weak binding between free FITC and gelatin microsphere. FITC was conjugated to bFGF molecules by a chemical bond, thus the fluorescent of GMSs in Fig 2 come from the encapsulated FITC-bFGF. The electrostatic interaction between bFGF and gelatin drive a homogeneous encapsulation of bFGF in GMSs, because of gelatin with an isoelectric point of 5.0 while bFGF with an isoelectric point of 9.6.

### The sustained-release of bFGF from bFGF-GMSs

**Fig 3:**
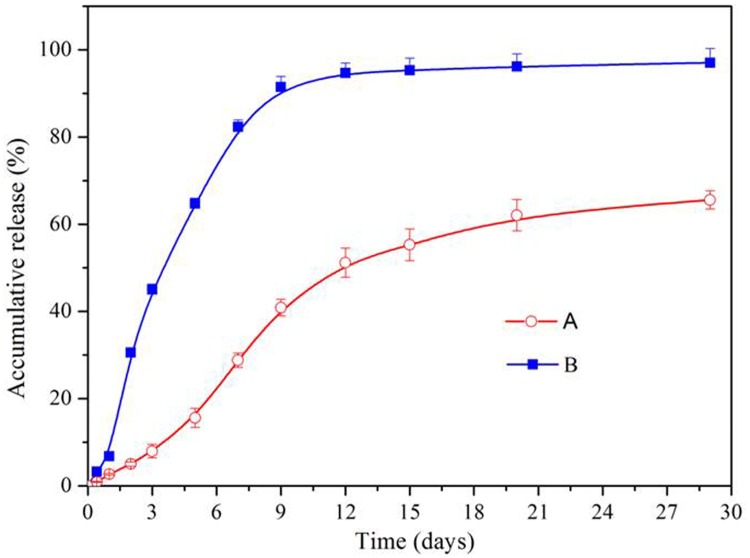
In vitro release of bFGF from porous microspheres. (A) and normal gelatin microspheres (B) (Three replicates of each point were done, mean value ±SD).

To evaluate the sustained-release profile of bFGF from microspheres, in vitro release study was conducted. The cumulative release of bFGF from microspheres was shown in Fig 3. The normal microspheres exhibited a rapid release profile with a rapid burst release, ca. 30% of the encapsulated bFGF releasing within the first 48 h, followed by a rapid complete release of bFGF over two weeks. In contrast, the porous microsphere displayed a continuous bFGF release pattern without an obvious burst release within 48h, followed by a slow release of bFGF over two weeks. Only ca. 65% of the encapsulated bFGF was released even after 28days. The different bFGF release profiles between normal microspheres and porous GMSs might be attributed to the different distribution of bFGF in the bulk microspheres. Generally, the burst drug release of microspheres was caused by the drug absorbed on the surface or localized near surface of microspheres [18]. Since the surface of normal microspheres was dense and of little porosity, thereby majority of bFGF may be adsorbed or localized near on the surfaces of microspheres, as shown in S2 Fig, resulting in a higher burst release. By contrast, the encapsulated bFGF in porous GMSs homogenously distributed inside the bulk porous microspheres without a significant adsorption on their surfaces, as shown in Fig 2D, making the diffusion pathway along which bFGF released from microsphere longer. Besides, the strong electrostatic interaction between acid gelatin and basic bFGF inside microspheres also resulted in its very slow release. All these factors resulted in the slow release behavior of bFGF from porous GMSs, remaining nearly 35% of its encapsulated bFGF in microspheres residues even after 28days release. The bioactivity of the released bFGF from the porous GMSs was tested using a proliferation assay of PC-12 cells. As shown in S4 Fig, the released bFGF over 30 days was bioactive and elicited PC12 cell proliferation compared with non-bFGF containing control medium.

### Behavioral change by different treatment

**Fig 4:**
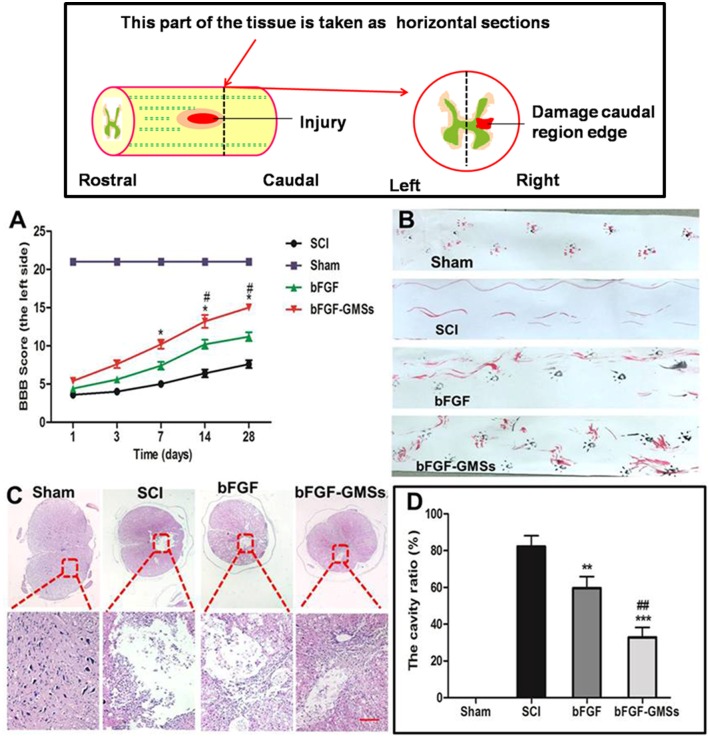
Behavioral improvement and cavity alteration. (A) BBB locomotion assessments of SCI rats. Distinguish between left foot and right foot. The score of sham group was 21 points, which means normal locomotion. (B) Footprint analyses of the different groups. (C) HE staining was carried out using hematoxylin and eosin dye. The nucleus is stained with blue and purple, and the cytoplasm is light red. (D) The size of the cavity is calculated and analysised by Image pro plus 7.0. Data are presented as Mean ± SEM, n = 5. *P<0.05, **P < 0.01, ***P < 0.001, #P < 0.05, ##P < 0.01 and ###P < 0.001. (n = 5). *: other groups VS SCI group, #: bFGF-GMSs group VS bFGF group. Scale bar = 100μm.

To compare the therapeutic effect of bFGF-GMSs and bFGF, SCI model rats were treated with bFGF-GMSs (20 μl, 60μg bFGF/each rat) or bFGF (20 μl, 60μg/each rat) by in situ injection. Behavioral recovery was evaluated using BBB rating scale and footprint recordings. The sham group obtained normal BBB scores at 21 score (Fig 4A). At 1 and 3 after contusion, there was no significant difference in BBB scores between bFGF-GMSs and bFGF, SCI group. Compared with the SCI group, the behavioral changes of rats became obvious in bFGF-GMSs group at the seventh day (*P*<0.05). At14 days and 28 days, bFGF-GMSs also show the advantages of treatment for motor function recovery of SCT tats (Fig 4A a&b). In the footprint analyses at 28 days, the rats treated with bFGF-GMSs had a fairly consistent coordination of hind limb and the toe dragging was not obvious (Fig 4B). The consistent coordination for the rats treated with free bFGF solution was not observed but the toe dragging was obvious. These indicated that GMSs could increase therapeutic effect of bFGF on functional improvement of rats after spinal cord injury.

### Histological changes

**Fig 5:**
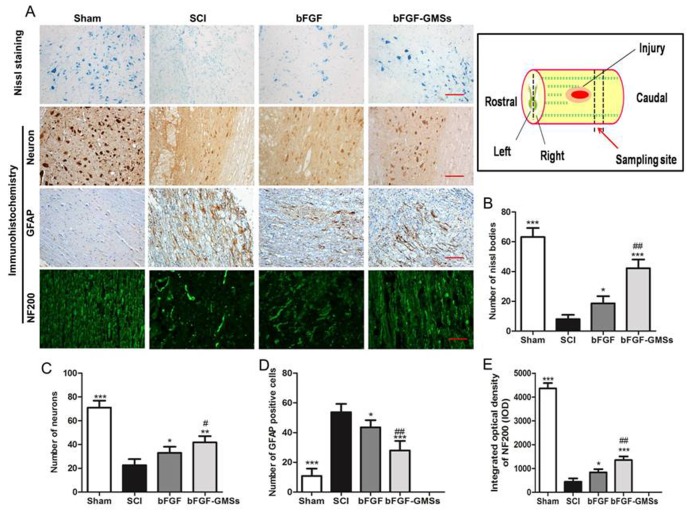
Histopathological changes. (A) Nissl bodies were stained with toluidine blue (nissl staining). Neurons, GFAP and NF200 in SCI rats was detected by immunohistochemical staining. The second antibody of NF200 is FITC luminescence antibody. Scale bar = 100μm (B) Numbers of nissl bodies was analyzed. (C) Analysis of numbers of neurons. (D) Analysis of the GFAP positive cells. (E) Optical density statistics of NF200. Data are presented as Mean ± SEM, n = 5. *P<0.05, **P < 0.01, ***P < 0.001, #P < 0.05, ##P < 0.01 and ###P < 0.001. (n = 5). *: other groups VS SCI group, #: bFGF-GMSs group VS bFGF group.

HE staining of injured spinal also revealed a progressive destruction of the dorsal white matter and central gray matter tissue and formed a cavity in the SCI group at caudal to the lesion after 28 days (Fig 5C). Compared with the SCI group, bFGF-GMSs group showed a significant protective effect with less necrosis, karyopyknosis, infiltrated polymorphonuclear leukocytes, and obviously reduced the cavity ratio (*P*<0.001, Fig 4D). This result further proved that GMSs could enhance neuroprotective effect of bFGF on SCI rats. The nissl bodies could reflect the regeneration of the axon [19]. Compared with the sham group, the SCI group exhibited the fewer nissl bodies, whereas they were increased after treatment with different bFGF-GMSs (*P* < 0.001) in Fig 5B, indicating the regeneration of injured axon.

**Fig 6:**
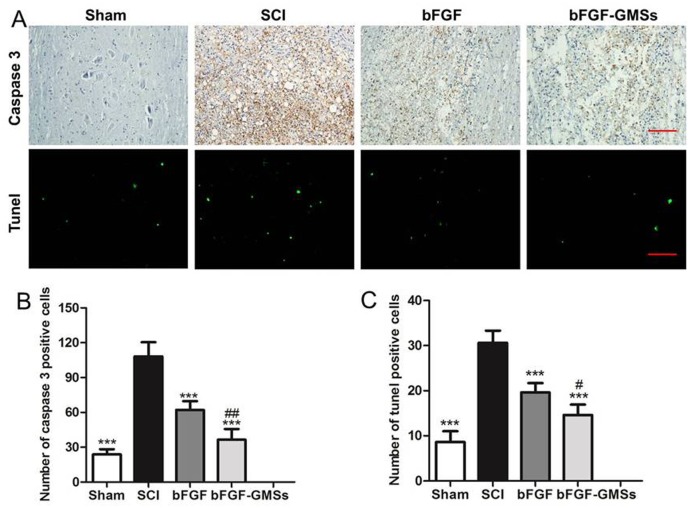
Apoptosis results invivo. (A) The expression of Caspase-3 in the immunohistochemical staining and the Tunel assay. Scale bar = 100μm. (B) Analysis of caspase 3 positive cells. (C) Analysis of Tunel apoptosis cell. *P<0.05, **P < 0.01, ***P < 0.001, #P < 0.05, ##P < 0.01 and ###P < 0.001. (n = 5). *: other groups VS SCI group, #: bFGF-GMSs group VS bFGF group.

To confirm the neuroprotective effect of bFGF-GMSs on SCI rat, the immunohistochemical staining of neurons, GFAP and NF200 in SCI rats were also detected after treatment, and the results were shown in Fig 6. The number of neuron decreased significantly in 28 days in SCI rats but it increased in SCI rats after treatment with the different bFGF formulations. Expectedly, the number of neuron in bFGF-GMSs group was more than that in the free bFGF solution group (*P*<0.05, Fig 5C). GFAP is a marker of astrocyte activation in the nervous system [20, 21]. Astrocytes usually promoted the formation of scar in chronic spinal cord injury, which hindered regeneration of axon. The results of GFAP staining indicated that bFGF reduced the expression of GFAP in SCI rats (*P*<0.05) and bFGF-GMSs has a more pronounced effect than the free bFGF (*P*<0.01, Fig 5D). NF200 was found in axons under normal conditions. The NF200-positive fibres in the SCI model rats were degraded, the axons were broken, and the arrangement disordered. After treatment with bFGF-GMSs, the disordered nerve filaments become regular and NF200 expression has significantly increased (*P*<0.05). Besides, the rostral part of the injury was also detected by staining GFAP and neuron. As shown in S5 Fig, whatever free bFGF or the encapsulated bFGF in GMSs inhibited the expression of GFAP in SCI rats (P<0.05). This result was similar to that of the caudal staining of the injury. However, tissue repair in the rostral part of the injury was not as good as the caudal part of the injury, because the tissue caudal to the injury was the most popular region representing the therapeutic effect, where stem cells transplantation may play an important role in facilitating recovery of injured spinal through inhibiting inflammation and polarization of M1 cells to the M2 state.

### Inhibition of apoptosis in vivo

Cell apoptosis plays a vital role in the progressive degeneration of injured spinal cord. The Caspase family played a very important role in the process of cell apoptosis. As a key executive molecule, caspase-3 initiated many cascades of apoptosis signal transduction. The immunofluorescent staining of caspase-3 was shown in Fig 6. Caspase-3 positive cells in the SCI group increased significantly at 28 d after SCI, in contrast with normal spinal cord. Moreover, the bFGF-GMSs exhibited the most effective inhibition of cell apoptosis (*P*<0.001, Fig 6B). Tunel assay was also carried out for further detection of cell apoptosis. Similar results were presented, while the number of positive cells at 28^th^ day after treatment with bFGF-GMSs was significantly reduced, and it was also fewer than that of the bFGF group (*P*<0.01, Fig 6C).

## Discussions

Spinal cord injury can lead to complete and permanent loss of neurologic function and be one of the most serious diseases of the nervous system (CNS). Spontaneous regeneration of the injured spinal cord is limited by numerous factors including an insufficient supply of growth promoting stimuli and an abundance of growth inhibitors. bFGF, a growth promoting stimulus, was secreted by fibroblast cells in body. Recently, many studies displayed that bFGF had multiple biological functions. It was firstly found be effective to induce the vascular growth factor and promote the wound healing and tissue repair of burned skin [22–24]. And then, it was found that bFGF was also an important stimulus to participate in regeneration and repairmen of the injured nerves [25].Our previous studies indicated that bFGF was potent to slow down the progression of brain stroke and parkinson through promoting nerve regeneration and tissue repair[3, 26, 27]. However, the half-life of b-FGF in vivo is very short, only about 1.5 minutes, and its biological activity is rapidly lost from the site of administration by diffusion and enzymatic degradation [28]. New delivery strategies and technologies have been developed to address these limitations. For example, nanoliposomes served as a carrier of bFGF, was administrated by the intranasal pathway to deliver bFGF toward the brain of a rat model with cerebral ischemia-reperfusion injury [3, 29]. Besides, local implantation of bFGF-containing biomaterial scaffolds into the spinal cord was also reported to be an effective strategy to overcome these issues [30].

In this study, we developed porous gelatin microspheres as a sustained-release platform of bFGF for local implantation into the injured spinal cord, promoting neural regeneration and recovery of neurologic function. Firstly, implantable porous gelatin microspheres were successfully prepared with non-toxic material, gelatin. Gelatin is biodegradable and biocompatible, which has been used as a nanoparticles-forming material for implantation in lung [26]. In order to avoid the organic solvents, a W/O emulsion template containing porous-forming agents was used to manufacture the porous microspheres. Existence of porosity on surface of microspheres allowed to easily encapsulating bFGF through immersing porous GMSs in the aqueous bFGF solution. The electrostatic interaction between bFGF and gelatin drive a homogeneous encapsulation of bFGF in microspheres, because of gelatin with an isoelectric point of 5.0 while bFGF with an isoelectric point of 9.6. The prepared microspheres have an optimal diameter with mean size of 35μm, smaller than the reported GMSs with diameter of ca.100μm in literature [6]. Therefore, bFGF-loaded GMSs were suitable for implantation by using a 16-gauge needle.

Furthermore, neuroprotective effect and morphologic recovery of the injured spinal were also evaluated in SCI rat model. Treatment with bFGF-GMSs also resulted in better result than that of bFGF. Firstly, the BBB score of the group treated with bFGF was much higher than that of the bFGF group, indicating better recovery of motor function. In view of the spinal cord injury on the right side, the activities of left hind limbs are commonly subject to constrain[31, 32]. At 7 days after treatment with bFGF-GMSs, the functional recovery of the left hind limb was more obvious and faster than that of free bFGF. Secondly, morphology of injured spinal cord was observed by HE staining. Rat treated with bFGF-loaded GMSs displayed a reduced cavity formation in comparison with free bFGF. The underlining molecular mechanisms of bFGF-loaded GMSs was involved in reducing activation of scar glial cells, nerve fibers and protecting neurons from further damage. Overall, when encapsulated in porous gelatin microspheres, the encapsulated bFGF maintained stable for a long time without loss of biological activity after implantation in injured spinal. Moreover, bFGF was slowly released from microspheres, which made bFGF-GMS generate better therapeutic result than bFGF.

Spinal cord injury is a complex pathological process causing motor sensation and disturbance [33]. Cellular apoptosis after SCI, the most common mechanism, plays an important role in neuron degeneration [34]. We found that treatment with bFGF-GMSs was more potent to inhibit cells apoptosis in comparison with bFGF. Expression of caspase-3, a key initiator protein of apoptosis pathway, was inhibited after treatment with bFGF-GMSs, indicating its anti-apoptosis. The neuroprotective effect for SCI was also confirmed by immunohistochemical staining of GFAP and NF200.

In summary, bFGF was encapsulated into implantable porous gelatin microspheres and then released in a sustained-release manner in injured spinal. These implantable bFGF-GMSs promoted healing of injured spinal in SCI rats, and accelerating recovery of neurologic function. Therefore, bFGF-GMSs may be a promising therapeutic approach for sustained-release of growth-promoting stimulus.

## Supporting information

S1 Fig SEM graphs of porous gelatin microspheres before washing. (DOC)

S2 Fig SEM graphs of normal gelatin microspheres without porous agent. (DOC)

S3 Fig Fluorescence microscopic graph of porous gelatin microspheres after loading free FITC. (DOC)

S4 Fig The bioactivity of the released bFGF from porous GMS. (DOC)

S5 Fig Neurons and GFAP in SCI rats were detected by immunohistochemical staining. (DOC)

S1 File Details about the care and use of animals and bioactivity assay for released b-FGF. (DOC)
