# Supplementary figures and images for: Implantable porous gelatin microspheres sustained release of bFGF and improved its neuroprotective effect on rats after spinal cord injury
Source: PLoS One. 2017 Mar 14;12(3):e0173814. doi: 10.1371/journal.pone.0173814 (PMC5349659; doi:10.1371/journal.pone.0173814)

**S1 Fig**. SEM graphs of porous gelatin microspheres before washing with water.


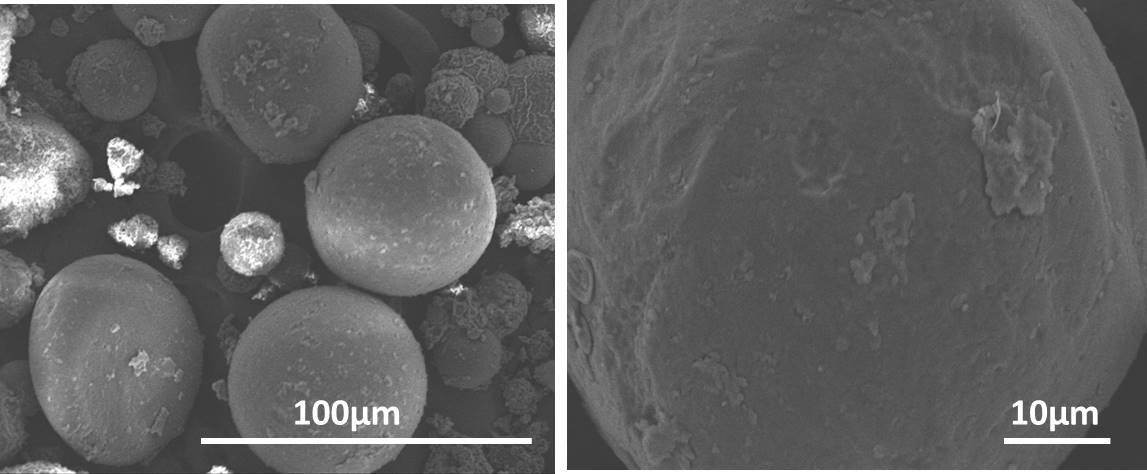

Supplement: S1 Fig — (DOC) [file pone.0173814.s001.doc]

**S3 Fig.** Fluorescence microscopic graph of porous gelatin microspheres after loading free FITC


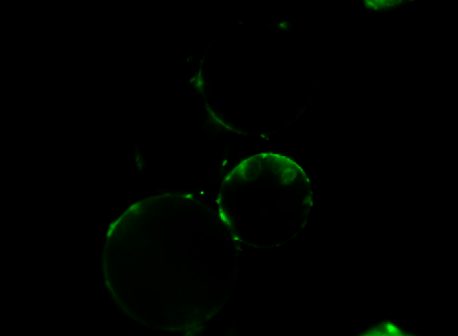

Supplement: S3 Fig — (DOC) [file pone.0173814.s003.doc]
