# Supplementary material for: Implantable porous gelatin microspheres sustained release of bFGF and improved its neuroprotective effect on rats after spinal cord injury
Source: PLoS One. 2017 Mar 14;12(3):e0173814. doi: 10.1371/journal.pone.0173814 (PMC5349659; doi:10.1371/journal.pone.0173814)

**S2 Fig.** (A) SEM graphs of normal gelatin microspheres without porous agent after wash with water, and (B) fluorescence microscopic graph of normal gelatin microspheres after loading FITC-bFGF (×10).


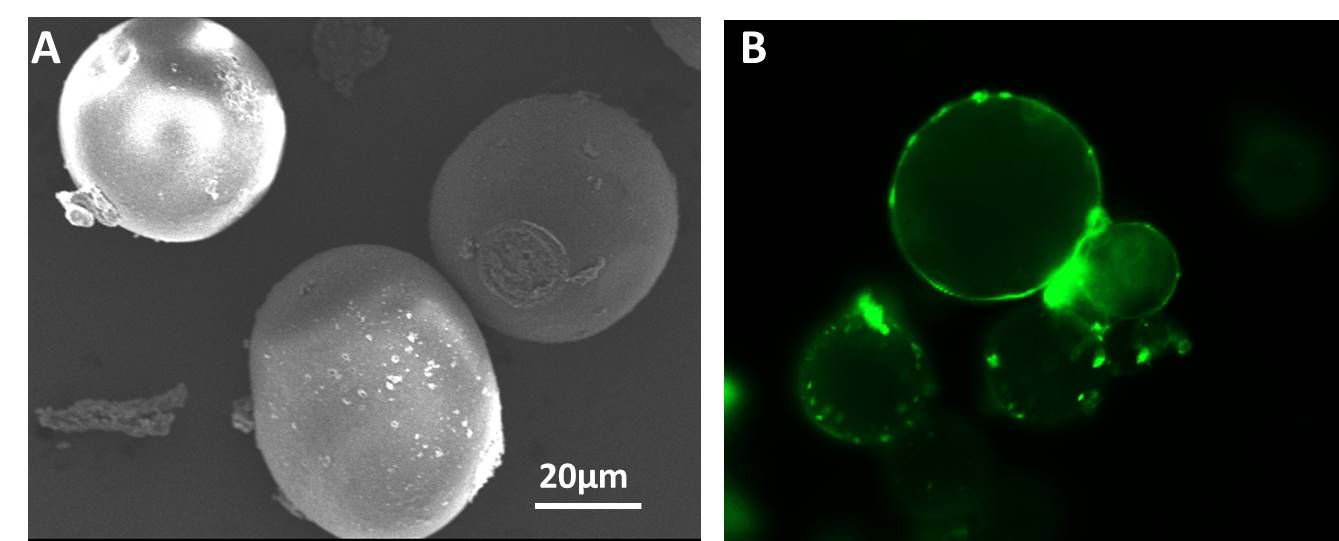

Supplement: S2 Fig — (DOC) [file pone.0173814.s002.doc]
