# Supplementary material for: Implantable porous gelatin microspheres sustained release of bFGF and improved its neuroprotective effect on rats after spinal cord injury
Source: PLoS One. 2017 Mar 14;12(3):e0173814. doi: 10.1371/journal.pone.0173814 (PMC5349659; doi:10.1371/journal.pone.0173814)

**S4 Fig**. The bioactivity of the released bFGF from porous GMS. The proliferation of PC12 cells was detected after incubation with fresh medium containing the released bFGF from bFGF-GMS; fresh medium without bFGF was used as negative control (No treatment). At 10^th^ days, the released bFGF had the strongest proliferative activity. This indicated that the maximum amount of bFGF was reached from 5^th^ to 10^th^ day. In the next few days, the proliferative activities gradually become weaken, because the amount of bFGF was decreased. Means ±standard deviations are reported. All samples were tested in triplicate. *treatment with bFGF-GMS VS No treatment, P<0.05.


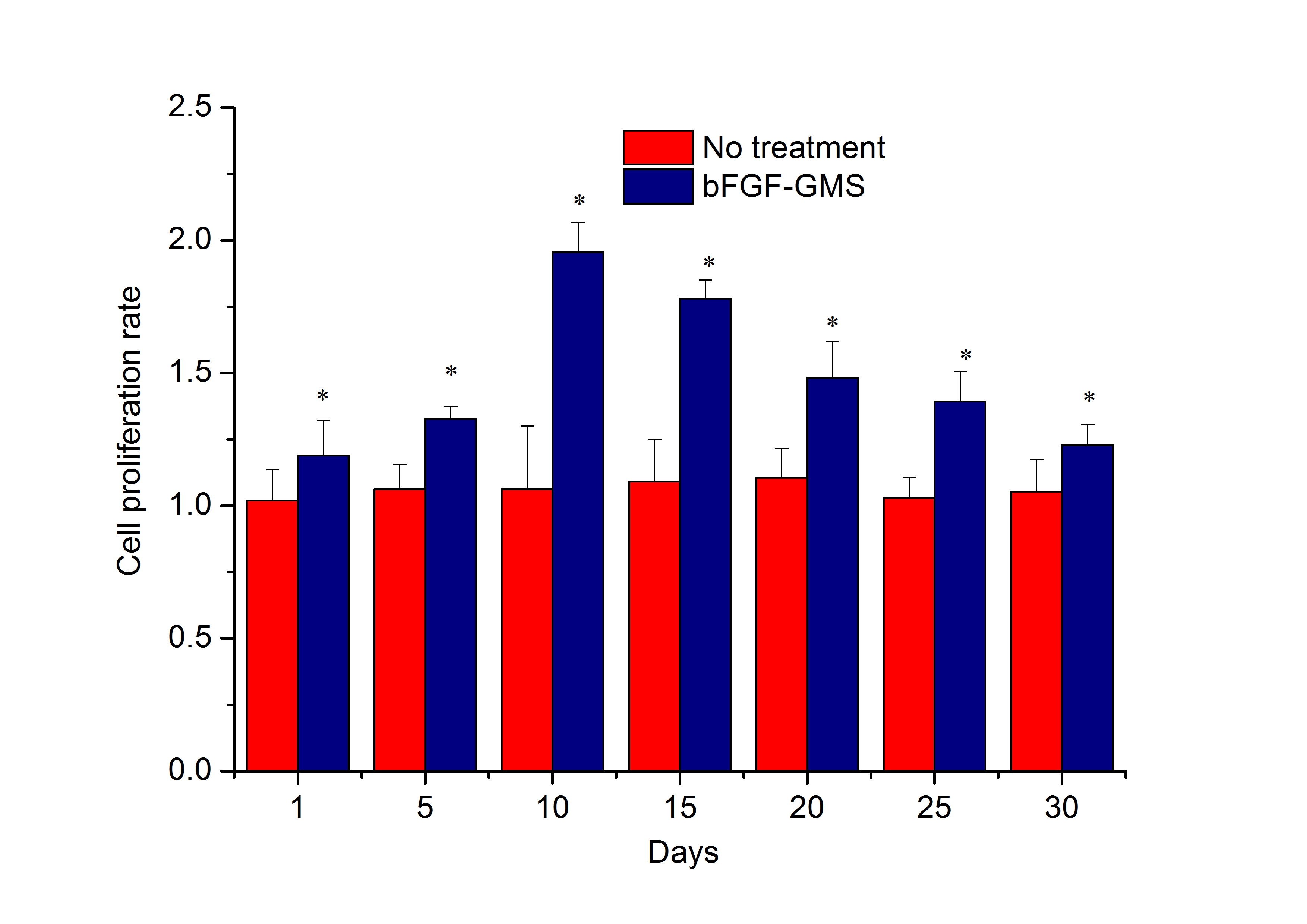

Supplement: S4 Fig — (DOC) [file pone.0173814.s004.doc]
