# Supplementary material for: Implantable porous gelatin microspheres sustained release of bFGF and improved its neuroprotective effect on rats after spinal cord injury
Source: PLoS One. 2017 Mar 14;12(3):e0173814. doi: 10.1371/journal.pone.0173814 (PMC5349659; doi:10.1371/journal.pone.0173814)

**S5 Fig**. Neurons and GFAP in SCI rats were detected by immunohistochemical staining on the rostral part of the injury (A). The analyzed numbers of neuron (B) and GFAP positive cells (C). Data are presented as Mean ± SEM, n=5. *P<0.05, ****P* < 0.001, #*P* < 0.05, (n=5). *: other groups VS SCI group, #: bFGF-GMSs group VS bFGF group.


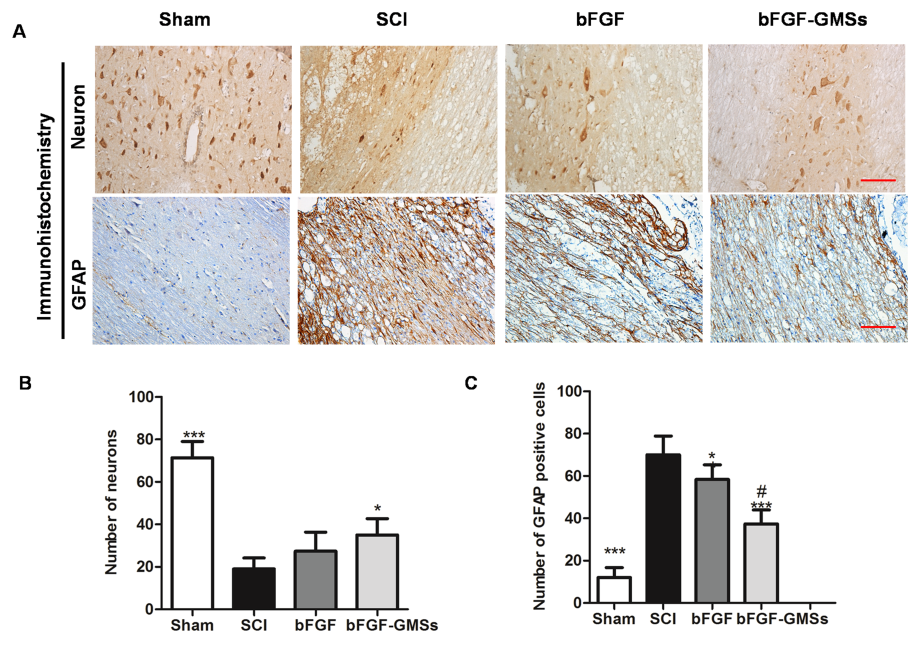

Supplement: S5 Fig — (DOC) [file pone.0173814.s005.doc]
