# Supplementary material for: Implantable porous gelatin microspheres sustained release of bFGF and improved its neuroprotective effect on rats after spinal cord injury
Source: PLoS One. 2017 Mar 14;12(3):e0173814. doi: 10.1371/journal.pone.0173814 (PMC5349659; doi:10.1371/journal.pone.0173814)
Supplement: S1 File — (DOC) [file pone.0173814.s006.doc]

**Details about the care and use of animals**

Sprague-Dawley rats (250 ± 20g, female, Slac Laboratory Animal Corporation, Shanghai, China) were housed at 25 ± 2 ℃ and humidity of 50±10% controlled with a 12 h light/12 h dark cycle, and given free access to water and food. Animal studies were performed with the approval from the ethical committee of Wenzhou Medical College and experiments were performed according to the National Institutes of Health Guide for the Care and Use of Laboratory Animals, so animals received high level of humane care. Before the experiment, the behavior of all rats was detected for adapting to the experiment detection. The Basso, Beattie, and Bresnahan scores and footprint analysis were implemented by reference standards (Acta Biomater. 2016 Jan; 29:71-80. Exp Neurol. 1982 Sep; 77(3):634-43.). All the animals were anaesthetized by an intraperitoneal injection of pentobarbital sodium (60 mg/kg, intraperitoneal), then placed on a constant temperature heating platform. Then surgical procedures were performed on deeply anesthetized animals. The experimental operation, which may cause pain and harm to animals, should be carried out carefully. After the experiment, the animals were kept in a cage to avoid cross effects. After surgery, rats were returned and received manual bladder expression twice daily until bladder function was restored. Before the animal tissues were acquired, rats were euthanasia by an intraperitoneal injection with pentobarbital sodium. The dose of euthanasia is about 3 times as much as the amount of narcotics. Criteria for judging the death of animals is continuous no spontaneous breathing for 2-3min and no blink reflex. (Good Laboratory Practice. Experimental methods and techniques of drug toxicology) The fresh tissue was fixed in 4% paraformldehyde to prepare for the later section processing. After the experiment, the animals were put into the bags and hand in the center of the school.

**Bioactivity assay for released b-FGF**

PC12 cells was used to evaluate the bioactivity of bFGF by quantifying cells proliferation using the CCK8 assay kit in response to the supernatant samples collected from the in vitro release experiments. PC-12 cells were cultured in high-glucose Dulbecco’s Modified Eagle’s Medium (DMEM) with 10% foetal bovine serum (FBS) and 1% penicillin-streptomycin in a humidified incubator containing 5% CO_2_ at 37 °C. The cells in their logarithmic growth phase were harvested with trypsin for further experiments. PC12 cells were cultured in a 96-well plate at a density of 5,000 cells per well for 24 hours. Then, the supernatant culture medium was removed and replaced with fresh medium containing the released bFGF. After 24 hours of incubation, 10 μl of CCK-8 solutions were added into the wells and incubated for 2 h to quantify cell proliferation. The absorbance was measured at 450 nm with a reference wavelength of 650 nm. The fresh medium without bFGF (No treatment group) was used as control sample.
